# Therapeutic Effects of PPAR***α*** on Neuronal Death and Microvascular Impairment

**DOI:** 10.1155/2015/595426

**Published:** 2015-01-29

**Authors:** Elizabeth P. Moran, Jian-xing Ma

**Affiliations:** ^1^Department of Cell Biology, University of Oklahoma Health Sciences Center, 941 Stanton L. Young Boulevard, BSEB 328B, Oklahoma City, OK 73104, USA; ^2^Department of Physiology, University of Oklahoma Health Sciences Center, 941 Stanton L. Young Boulevard, BSEB 328B, Oklahoma City, OK 73104, USA; ^3^Department of Medicine, University of Oklahoma Health Sciences Center, 941 Stanton L. Young Boulevard, BSEB 328B, Oklahoma City, OK 73104, USA; ^4^Harold Hamm Diabetes Center, University of Oklahoma Health Sciences Center, 941 Stanton L. Young Boulevard, BSEB 328B, Oklahoma City, OK 73104, USA

## Abstract

Peroxisome-proliferator activated receptor-alpha (PPAR*α*) is a broadly expressed nuclear hormone receptor and is a transcription factor for diverse target genes possessing a PPAR response element (PPRE) in the promoter region. The PPRE is highly conserved, and PPARs thus regulate transcription of an extensive array of target genes involved in energy metabolism, vascular function, oxidative stress, inflammation, and many other biological processes. PPAR*α* has potent protective effects against neuronal cell death and microvascular impairment, which have been attributed in part to its antioxidant and anti-inflammatory properties. Here we discuss PPAR*α*'s effects in neurodegenerative and microvascular diseases and also recent clinical findings that identified therapeutic effects of a PPAR*α* agonist in diabetic microvascular complications.

## 1. Introduction

### 1.1. Peroxisome-Proliferator Activated Receptor-Alpha (PPAR*α*)

PPAR*α* is a transcription factor and belongs to the nuclear receptor superfamily [1]. PPAR*α* is activated when bound by endogenous lipid/lipid metabolite ligands or synthetic xenobiotic ligands [2]. Once activated, PPAR*α* heterodimerizes with the Retinoid X Receptor (RXR) and binds to PPAR Response Elements (PPREs) in the promoter regions of target genes involved in diverse processes such as energy metabolism, oxidative stress, inflammation, circadian rhythm, immune response, and cell differentiation [3–8]. PPAR*α* has beneficial effects in many diseases but also plays a pathological role in some conditions, for example the development of insulin resistance [3].

PPAR*α* has neuroprotective effects in several disease models including stroke, Alzheimer's disease, Parkinson's disease, traumatic brain injury, diabetic peripheral neuropathy, and retinopathy [8–12]. These neuroprotective effects have been attributed largely to PPAR*α*'s antioxidant and anti-inflammatory properties, although its beneficial effects in lipid metabolism and glucose homeostasis may also play a role [7–11].

PPAR*α* also has beneficial effects in the vasculature and plays a more prominent role in the microvasculature than in the macrovasculature. PPAR*α* has protective effects in endothelial dysfunction, hypertension, vasoregression, pathological neovascularization, and vascular hyperpermeability [13–15]. These effects are also modulated by decreased oxidative stress and inflammation and additionally increased endothelial nitric oxide synthase (eNOS) activation, improved endothelial function, and decreased levels of vascular growth factors.

Interestingly, PPAR*α* is downregulated in the diabetic retina and kidney, and although the regulatory mechanisms responsible for diabetes-induced PPAR*α* downregulation are unclear, decreased PPAR*α* levels may play a pathological role in diabetic microvascular complications [15, 16]. Further, our group found that retinal levels of PPAR*α*, but not PPAR*γ* or PPAR*β*/*δ*, were decreased in diabetes, suggesting that PPAR*α* plays a more crucial role than other PPARs in repressing development of diabetic retinopathy (DR) [15].

Two major clinical trials have evaluated the effects of the PPAR*α* agonist in diabetic complications and identified as tertiary outcomes that fenofibrate significantly decreased diabetic microvascular complications including retinopathy, nephropathy, and peripheral neuropathy in type 2 diabetic patients [17, 18]. These tertiary outcomes were identified by intent to treat analysis, leaving the underlying physiological and molecular mechanisms of action incompletely understood. PPAR*α* has since become a topic of intense investigation in diabetic microvascular complications [2].

### 1.2. Neuronal Cell Death

In neuronal cell death, neurons of the central or peripheral nervous systems die due to age-related conditions, traumatic injury, diabetic insults, vascular dysfunction, ischemia, metabolic aberrations, or a combination of these and other factors [40–43]. Although the molecular pathogenesis for neurodegenerative disease is unique to each condition, oxidative stress, inflammation, and microvascular dysfunction play prominent roles in many neurodegenerative diseases, and interventions that correct these parameters have therapeutic effects [40–42].

### 1.3. Microvascular Impairment

Microvascular aberrations participate in the pathogenesis of myriad diseases, and interventions for these abnormalities have considerable therapeutic potential. Endothelial dysfunction, vascular hyperpermeability, pericyte dropout, vasoregression, and neovascularization play prominent roles in microvascular disease [44–46]. The molecular mechanisms for these abnormalities are complex, but inflammation, oxidative stress, vascular growth factors, dyslipidemia, and tight junction interruption are major contributing factors [44–46]. Further, neurodegeneration may also cause microvascular impairment in some neurovascular diseases, such as DR and ischemic stroke.

## 2. Protective Effects of PPAR*α* in Neuronal Cell Death

PPAR*α* has neuroprotective effects in many disease models including cerebral ischemia/reperfusion, traumatic brain and spinal cord injury, Parkinson's disease, Alzheimer's disease, peripheral neuropathy, ischemic retinopathy, and DR (Table 1). PPAR*α*'s neuroprotective capacity has been largely attributed to its antioxidant and anti-inflammatory effects, which may decrease neuronal cell death in these models [9, 28]. However, PPAR*α*'s beneficial effects in endothelial survival and function may also play a role in PPAR*α*-mediated neuroprotection, as vascular dysfunction contributes to many neurodegenerative diseases [47, 48].

The molecular basis of neuronal cell death is complex and may be context dependent. However, oxidative stress and inflammation play prominent roles in many neurodegenerative diseases, and experimental evidence suggests that PPAR*α*'s antioxidant and anti-inflammatory properties may be responsible in part for its neuroprotective effects.

Although physiological reactive oxygen species (ROS) levels play critical roles in cellular signaling and physiology [49], an overabundance of ROS may be detrimental. ROS are highly unstable intermediates, and oxidize cellular macromolecules such as phospholipids, proteins, and DNA [50]. This oxidative damage, or oxidative stress, leads to cellular death and dysfunction, including neurodegeneration [50]. Oxidative stress also increases inflammation, glial activation, and mitochondrial dysfunction, further exacerbating neurodegeneration. Neurons are acutely sensitive to ROS, and oxidative stress contributes to neurodegeneration in many disease models.

Neuronal inflammation, or neuroinflammation, plays a significant role in neurodegenerative disease. Inflammation in neuronal cells directly activates apoptotic pathways through mitogen activated protein kinase (MAPK) and nuclear factor kappa-light-chain-enhancer of activated B cells (NF-*κ*B) signaling [51]. Neuroinflammation also results in endothelial cell (EC) loss, blood-brain barrier breakdown, and glial activation, further exacerbating neurodegeneration.

### 2.1. Cerebral Ischemia

Deplanque et al. first demonstrated that PPAR*α* is neuroprotective in cerebral ischemia [9]. The authors subjected wild-type and apolipoprotein E-deficient (*ApoE* ^−/−^) mice to middle cerebral artery occlusion and identified that the PPAR*α* agonist fenofibrate decreased the susceptibility of* ApoE* ^−/−^ mice to stroke and also decreased infarct volume in wild-type animals [9]. These effects were abrogated by PPAR*α* ablation, indicating that fenofibrate's neuroprotective effects in this model were PPAR*α*-dependent [9]. The same study identified that fenofibrate significantly increased the activities of antioxidant enzymes superoxide dismutase and catalase in cerebral ischemia, which deactivate ROS to alleviate oxidative stress [9]. Further, PPAR*α* decreased expression of vascular adhesion molecules, subsequently lessening inflammation and improving vasoreactivity in animals subjected to middle cerebral artery occlusion [9]. The authors thus concluded that PPAR*α*'s neuroprotective effects in cerebral ischemia were due to alleviation of ischemia-induced oxidative stress and inflammation and improved cerebral microvascular function [9].

Ouk et al. also identified that fenofibrate had a neuroprotective effect in ischemic brain injury by subjecting rats and mice to midcerebral artery occlusion and measuring infarct volume, motor and cognitive function, vascular function, and neurogenesis [19]. Fenofibrate improved neuronal function and decreased infarct volume in acute cerebral ischemia and also improved vascular response [19]. Additionally, fenofibrate modulated neurorepair and inhibited the amyloid cascade, suggesting that it may have protective effects in other traumatic brain injury models and chronic neurodegenerative diseases [19].

Other studies have also demonstrated that PPAR*α* improves outcomes of cerebral ischemia and that its protective effects may be due to its antioxidant and anti-inflammatory properties and beneficial effects in vascular function, which may be through similar molecular mechanisms to those described above [20, 52].

### 2.2. Traumatic Brain and Spinal Cord Injury

PPAR*α* has neuroprotective effects in traumatic brain and spinal cord injury, which are modulated by its anti-inflammatory and antioxidant effects [21, 22]. Genovese et al. subjected wild-type and PPAR*α* knockout (*PPARα* ^−/−^) mice to spinal cord compression injury and observed that spinal cord trauma, neutrophil infiltration, oxidative stress, and neuronal apoptosis were significantly increased in* PPARα* ^−/−^ mice in comparison to wild-type mice [21]. Further, Besson et al. subjected rats to traumatic brain injury and demonstrated that fenofibrate improved neurological deficit, brain lesions, and cerebral oedema and decreased intracellular adhesion molecule-1 (ICAM-1) expression, suggesting neuroprotective and anti-inflammatory effects, although the precise molecular mechanisms of action were not defined [22]. Other studies have also suggested that PPAR*α* agonists have neuroprotective effects in similar models [23–25].

### 2.3. Alzheimer's Disease

Clinical and basic research findings have suggested that PPAR*α* may have a therapeutic effect in Alzheimer's disease, but these findings remain controversial. van Rossum and Hanisch identified that PPAR*α* agonists inhibited beta-amyloid stimulated proinflammatory responses* in vitro*, and Santos et al. demonstrated that PPAR*α* had a protective effect against beta-amyloid-induced neurodegeneration [52, 53]. However, Kukar et al. found that fenofibrate increased beta-amyloid production* in vitro*, although this interaction was not demonstrated to be PPAR*α*-dependent, so it may be an off-target effect [54]. A genetic epidemiological study suggested that PPAR*α* single nucleotide polymorphisms (SNPs) were associated with increased Alzheimer's disease risk, although later studies contradicted this finding [55, 56].

### 2.4. Parkinson's Disease

Recent Studies have demonstrated that PPAR*α* holds potential as a therapeutic target for Parkinson's disease, which is a chronic neurodegenerative disorder of the central nervous system characterized by loss of dopaminergic neurons [57].

Fenofibrate and PPAR*α* had neuroprotective effects in a toxin-induced model of Parkinson's disease, and these effects were mediated in part by decreased oxidative stress [8, 26]. Barbiero et al. also demonstrated that PPAR*α* and PPAR*γ* agonists had protective effects in a similar animal model of Parkinson's disease, preserving locomotor and cognitive activity and preventing loss and dysfunction of dopaminergic neurons [58].

Uppalapati et al. corroborated that fenofibrate was neuroprotective in Parkinson's disease and suggested that this effect was due to decreased inflammation in the brains of fenofibrate-treated animals [27]. Importantly, this study also used pharmacokinetic analysis to demonstrate that fenofibric acid, the bioactive metabolite of fenofibrate, was present in the brains of fenofibrate-treated animals, suggesting that fenofibrate was metabolized and successfully crossed the blood-brain barrier* in vivo* [27].

Although the mechanism(s) of action for PPAR*α*-mediated neuroprotection in Parkinson's disease have not been fully defined, Barbiero et al. found that fenofibrate-treated animals had decreased levels of oxidative stress biomarkers, suggesting an antioxidant effect [8, 58]. Further, Uppalapati et al. found that fenofibrate decreased brain levels of proinflammatory mediators, suggesting that PPAR*α* also has anti-inflammatory effects in this model [27].

### 2.5. Peripheral Neuropathy

The Fenofibrate Intervention and Event Lowering in Diabetes (FIELD) clinical trial identified that fenofibrate significantly decreased diabetic peripheral neuropathy (DPN) in human patients, as demonstrated by decreased nontraumatic limb amputation and improved sensory threshold in patients receiving fenofibrate treatment [11, 29]. Cho et al. have since revealed that fenofibrate has a therapeutic effect in DPN in a mouse model of type 2 diabetes and may modulate this effect in part by improving endothelial and neuronal survival through AMP-activated protein kinase (AMPK)/phosphoinositide-3 kinase (PI3K) activation [28]. Although the downstream antiapoptotic mechanisms for PI3K are not evaluated in the experimental model, the authors propose that inhibition of MAPK signaling and caspase activity together with increased expression of the antiapoptotic proteins survivin and Bcl-2 may be responsible for fenofibrate's cytoprotective effects in DPN [28].

Basic research findings have also demonstrated that PPAR*α* has a protective role in neuropathic pain, although the mechanisms for these effects are not fully understood. Ruiz-Medina et al. demonstrated that* PPARα* ^−/−^ mice were more susceptible to visceral and acute thermal nociception and had higher levels of proinflammatory factors in sciatic nerve injury [59]. Additionally, PPAR*α* agonists have analgesic effects in visceral, inflammatory, and neuropathic pain [60–62].

### 2.6. Retinopathy

Because PPAR*α* has a therapeutic effect in DR and is neuroprotective in several disease models, it is reasonable to hypothesize that PPAR*α* may be neuroprotective in retinopathy, which is characterized in part by neurodegeneration [4, 63, 64]. We and others have demonstrated that PPAR*α* has neuroprotective effects in retinopathy and that this protective effect may be due to alleviation of oxidative stress and inflammation [12, 30].

Our group first demonstrated that activation and expression of PPAR*α* had a neuroprotective effect in oxygen-induced retinopathy (OIR), a model of ischemic retinopathy [12]. In contrast, PPAR*α* ablation exacerbated ischemia-induced neuron death. In OIR, PPAR*α* repressed activation of hypoxia-inducible factor-1-alpha (HIF-1*α*) and subsequently decreased HIF-1*α*-driven transcription of NADPH oxidase-4 (Nox4), which produces ROS by catalyzing electron transport from NADPH to molecular oxygen [12, 65]. Further, PPAR*α* inhibited hypoxic ROS production* in vitro*, and we suggested that this effect was due to decreased Nox4 levels [12]. We postulate that this antioxidant effect may be responsible in part for PPAR*α*-mediated neuroprotection in retinal ischemia [12].

Similarly, Bogdanov and colleagues identified that fenofibrate had a neuroprotective effect in DR using db/db mice, a model of type 2 diabetes [30]. The authors demonstrated that electroretinogram (ERG) amplitude declined in diabetic mice and was improved by fenofibrate [30]. In the same model, retinal glial activation was increased in DR and partially decreased by fenofibrate [30]. Although this study did not define the molecular mechanisms of action, the authors propose that fenofibrate may confer neuroprotection in DR by alleviating inflammation and/or oxidative stress in the diabetic retina [30].

## 3. Beneficial Effects of PPAR*α* in Microvascular Impairment

PPAR*α* is well known for its beneficial effects in the microvasculature, and clinical trials have demonstrated that it has potent therapeutic effects in diabetic microvascular complications [17, 18]. Further, decreased PPAR*α* levels in diabetes are thought to contribute to inflammation, vascular damage, and neurodegeneration, and exogenous PPAR*α* agonists may compensate for this effect [15]. PPAR*α*'s beneficial effects are multifaceted, and PPAR*α* downregulation has been found to play important roles in vasoregression, endothelial dysfunction, vascular hyperpermeability, and pathological angiogenesis (Table 2).

### 3.1. Vasoregression

Vasoregression plays a prominent role in many microvascular diseases, particularly in the central and peripheral nervous systems. In vasoregression, EC and pericyte apoptosis, or dropout, results in tissue nonperfusion, which is particularly detrimental to metabolically demanding and highly sensitive neuronal tissues [66–68]. EC apoptosis plays a role in peripheral neuropathy, stroke, traumatic brain injury, and retinopathy [69–71]. In retinopathy, vasoregression-related ischemia also leads to overcompensatory, sight-threatening pathological neovascularization (NV) characteristic of proliferative retinopathies [72].

Vasoregression is a multifaceted process, but EC/pericyte dropout has been attributed in part to ischemia, oxidative stress, inflammation, and endothelial dysfunction [69]. In addition to EC and pericyte apoptosis, reparative endothelial progenitor cells (EPCs), which replace apoptotic ECs and secrete beneficial growth factors, may be compromised in some disease conditions, such as diabetes, further contributing to vasoregression and vascular dysfunction [73, 74].

Our group demonstrated in type 1 diabetic models that fenofibrate and PPAR*α* had a protective effect against DR-induced EC and pericyte dropout, decreasing acellular capillary formation and pericyte loss in the retinas of diabetic animals [13, 15]. In these models, PPAR*α* alleviated oxidative stress and inflammation by suppressing NF-*κ*B activation and subsequent transcription of Nox4 and inflammatory mediators, thereby decreasing oxidative stress and inflammation, respectively [13, 15]. Cho et al. also demonstrated that PPAR*α* decreased EC loss in peripheral diabetic neuropathy and suggested that this effect was mediated in part through AMPK activation and resultant activation of downstream cytoprotective pathways and improvements in endothelial function and vasorelaxation [28].

Further, Deplanque et al. identified that fenofibrate decreased EC loss in a rodent model of cerebral ischemia and suggested that this effect was due in part to increased activity of antioxidant enzymes superoxide dismutase and catalase, with subsequent alleviation of ischemia-related oxidative stress [9]. These findings were further supported in other rodent models of cerebral ischemia and related disorders [19, 20].

Because EC loss and subsequent vasoregression contribute to neurodegeneration in cerebral ischemia, DR, peripheral neuropathy, and age-related neurodegenerative diseases [75], it is likely that PPAR*α*-mediated vasoprotection contributes to the observed neuroprotective effects in these models.

### 3.2. Endothelial Dysfunction

Endothelial function is regulated by vasoactive factors that maintain proper vascular wall tone to regulate blood flow and prevent vascular inflammation [76]. Nitric oxide (NO) is a potent vasodilator and is necessary for endothelial function [77]. In diabetes and other pathological conditions, the production and bioavailability of NO are compromised, leading to a persistent state of vasoconstriction, inflammation, and oxidative stress [77, 78]. Endothelial dysfunction plays a prominent role in microvascular disease, limiting blood flow and increasing inflammation and oxidative stress [46, 79].

Clinical studies have demonstrated that in human diabetic patients, fibrates decrease markers of endothelial dysfunction and have beneficial effects in vascular function [31–34, 80–82]. These beneficial effects may be mediated in part by increased activation and production of eNOS, decreased endothelin-1 expression, and deactivation of inflammatory NF-*κ*B signaling, subsequently increasing NO levels and alleviating inflammation to improve endothelial function [14, 83, 84]. We and others have also demonstrated that PPAR*α* has beneficial effects in the diabetic microvasculature, and these effects may be due in part to decreased endothelial dysfunction in diabetic conditions [15, 28, 35].

Endothelial dysfunction also plays a prominent role in neurodegenerative disease [66, 85, 86]. It is therefore likely that PPAR*α* restoration of endothelial dysfunction may be responsible in part for its neuroprotective effects in these diseases.

### 3.3. Vascular Hyperpermeability

Increased vascular permeability, or vascular hyperpermeability, plays a role in diabetic complications, cerebral ischemia, heart failure, and many other diseases [87–89]. Vascular hyperpermeability is caused by EC dropout, inflammation, increased vascular growth factors, and EC tight junction dysfunction [90, 91]. Increased vascular permeability decreases the efficiency of the vasculature and results in widespread ischemia [46]. Vascular hyperpermeability also increases inflammatory processes such as leukostasis and may allow leukocyte infiltration [46].

Our group has identified that activation and expression of PPAR*α* decreases retinal vascular hyperpermeability in animal models of type 1 diabetes and ischemic retinopathy [15, 35]. We have also demonstrated in previous studies that PPAR*α* protects against pericyte and EC dropout in DR, suggesting that PPAR*α* inhibition of vascular hyperpermeability may be due in part to its protective effects against vasoregression [13].

Mazzon et al. also established that PPAR*α* improves tight junction integrity in an animal model of stress-induced intestinal permeability [37]. The authors identified that in* PPARα* ^−/−^ animals, intestinal permeability was significantly increased under restraint stress [37]. Further, mislocalization of tight junction proteins was increased in* PPARα* ^−/−^ mice, suggesting that PPAR*α* modulates small intestinal tight junction integrity.

Further, several studies have also suggested that PPAR*α* attenuates blood-brain barrier disruption in HIV-induced cerebrovascular toxicity and cerebral ischemia [92–94]. In HIV-induced cerebrovascular toxicity, PPAR*α* improves HIV deregulation of tight junction proteins by modulating matrix metalloproteinase and proteasome activities, subsequently alleviating tight junction disruption and vascular hyperpermeability in the model [92]. Although PPAR*α*'s beneficial effects upon the blood-brain barrier in cerebral ischemia are not fully understood [94], PPAR*α* may also improve tight junction integrity in this model through mechanisms similar to that in intestinal permeability and HIV-induced cerebrovascular toxicity.

Vascular hyperpermeability and disruption of the blood-brain barrier also play a role in other neurodegenerative diseases, such as Alzheimer's disease, Parkinson's disease, and traumatic brain injury [95–97]. It is thus possible that PPAR*α*'s identified therapeutic effects in these diseases are due in part to improved blood-brain barrier function, which may be modulated through restoration of tight junction proteins or alleviation of inflammation and/or oxidative stress.

### 3.4. Neovascularization

Pathological NV plays a central role in many diseases including proliferative retinopathies, tumor angiogenesis, atherosclerosis, and others [98–100]. The physiological and molecular mechanisms for NV are complex but are modulated in part by ischemia, inflammation, oxidative stress, and vascular growth factors [45, 101–103]. PPAR*α* is able to repress pathological angiogenesis in part by decreasing inflammation, oxidative stress, and vascular growth factor levels.

We previously demonstrated that PPAR*α* inhibited NV in an OIR model of ischemic retinopathy [35]. Our findings suggested that PPAR*α* decreased expression of vascular endothelial growth factor (VEGF) and its receptors, potentially by deactivating pathological Wnt signaling in retinopathy [15, 35]. It is also possible that PPAR*α*-mediated neuroprotection and/or vasoprotection may be responsible in part for PPAR*α*'s repression of retinal NV [12, 13, 36].

Additionally, Varet et al. identified that fenofibrate repressed angiogenesis in a nude mouse model and in an* in vitro* wound healing assay and suggested that PPAR*α* may deactivate Akt signaling to inhibit EC proliferation in these models [38]. Meissner et al. also found that PPAR*α* repressed VEGF receptor 2 (VEGFR2) expression by repressing specificity protein 1 (Sp1) binding to the VEGFR2 promoter in human vascular endothelial cells (HUVECs), thereby decreasing VEGF signaling [39].

EPCs play a protective role in vasoregression, but may also contribute to pathological NV, particularly in proliferative retinopathies. In some disease conditions, EPCs also shift to a proinflammatory phenotype that promotes NV [104]. In pathological NV conditions, EPCs migrate to neovascular areas and incorporate themselves into the neovasculature and also secrete vascular growth factors and inflammatory mediators that further exacerbate NV [104].

Our group identified that PPAR*α* suppressed bone marrow EPC mobilization in OIR, a mouse model of ischemic retinopathy [36]. We demonstrated in this study that PPAR*α* decreased retinal expression of EPC homing factors erythropoietin (Epo) and stromal-derived factor-1 (SDF-1) by suppressing HIF-1*α* activation, therefore inhibiting bone marrow-derived EPC release and homing to the retina [36]. It is thus feasible that PPAR*α* suppression of pathological EPC release may contribute to antiangiogenic effects identified in previous studies.

## 4. Clinical Findings

PPAR*α* has been identified as an attractive therapeutic target for diabetic complications, and most clinical studies of PPAR*α* have focused predominantly upon its potential therapeutic effects in diabetic complications. The fibrates, a class of lipid-lowering drugs designed to activate PPARs, are the most commonly used PPAR agonists clinically. Fenofibrate in particular is well tolerated and unlike other fibrates does not compete with statins for hepatic clearance, so is utilized nearly exclusively to treat dyslipidemic diabetic patients [105].

Two large randomized perspective clinical trials have demonstrated that fenofibrate decreases the prevalence of diabetic microvascular complications in human patients [18, 63]. The Fenofibrate Intervention and Event Lowering in Diabetes (FIELD) trial first identified that fenofibrate monotherapy had a therapeutic effect in DR, neuropathy, and nephropathy, and the Action to Control Cardiovascular Risk in Diabetes (ACCORD) trial later demonstrated that fenofibrate in a simvastatin background also had therapeutic effects in diabetic microvascular complications [18, 63].

### 4.1. FIELD Study

The FIELD study was conducted principally to evaluate fenofibrate's potential therapeutic effects in type 2 diabetes-associated cardiovascular disease [17]. Nearly 10,000 persons with type 2 diabetes from 50 to 75 years old were treated with either fenofibrate or a placebo for five years, and primary outcomes of coronary heart disease or nonfatal myocardial infarct were evaluated by intent to treat analysis [17]. Total cardiovascular events were analyzed as a secondary outcome, and microvascular complications were a tertiary outcome of the FIELD study [17].

Fenofibrate did not change total coronary events, but modestly decreased total cardiovascular events [17]. It is possible, however, that increased statin use by placebo-allocated patients may have masked fenofibrate's beneficial effects for this outcome [17]. Conversely, fenofibrate had a dramatic therapeutic effect in microvascular diabetic complications, significantly decreasing the incidence of retinopathy, nephropathy, and neuropathy [11, 17, 63, 106]. Because microvascular complications were a tertiary outcome and were identified by intent to treat analysis, the physiological and molecular mechanisms of action for these therapeutic effects were largely unknown when the FIELD trial findings were published [17, 63].

Interestingly, although fenofibrate's primary clinical application is dyslipidemia, FIELD participants' lipid profiles were modestly affected by fenofibrate [17]. Fenofibrate decreased serum triglycerides by approximately 30%, but this beneficial effect did not directly correlate with fenofibrate's therapeutic effects in microvascular complications [17, 63]. These findings suggest that fenofibrate's therapeutic effects in diabetic microvascular complications may be due in part to lipid-independent mechanisms, as has been further confirmed by the basic research findings outlined above.

### 4.2. ACCORD Lipid Study

The ACCORD study was conducted to evaluate the effects of intense glycemic control, hypertensive control, and combination lipid therapy upon cardiovascular disease risk in type 2 diabetes. The ACCORD Lipid trial was unique from the FIELD trial in that patients received fenofibrate in a statin background as opposed to fenofibrate monotherapy.

Similar to the FIELD trial, the ACCORD trial also identified that fenofibrate did not affect total coronary events but did decrease incidence of nonfatal myocardial infarct [64]. Fenofibrate also had a therapeutic effect in diabetic microvascular complications including nephropathy, retinopathy, and nontraumatic limb amputation [18].

Together these studies suggested that PPAR*α* had significant therapeutic potential in diabetic microvascular complications but gave little insight into the physiological and molecular mechanisms responsible for its therapeutic effects. PPAR*α* has since been a topic of intense investigation for diabetic microvascular disease, and several basic research studies have begun to delineate its effects in DR, neuropathy, and nephropathy [13, 15, 28, 35, 107, 108].

## 5. Conclusions and Future Directions

Both clinical and basic research findings have suggested that PPAR*α* has robust neuroprotective and vascular homeostatic effects. These beneficial effects may be due in part to PPAR*α*'s anti-inflammatory and antioxidant properties and also to restoration of endothelial function and vascular tight junction integrity. PPAR*α*'s abilities to decrease oxidative stress, inflammation, and endothelial dysfunction resulting from a variety of pathophysiological events undoubtedly play significant roles in its therapeutic effects.

However, because PPAR*α* target genes are diverse, it is likely that many other mechanisms contribute to both its beneficial and pathological effects in these and other disease models. Ongoing research efforts seek to broaden these horizons to better understand PPAR*α*'s systemic, whole organism effects.

## Figures and Tables

**Table 1:** Neuroprotective effects of PPAR*α* and molecular mechanisms of action.

Model	Physiological effects	Molecular mechanism(s)	Ref(s)
Cerebral ischemia	↓ Neuron loss ↓ Infarct volume	Antioxidant Anti-inflammatory ↓ Amyloid cascade	[9, 19, 20]

Traumatic brain/spinal cord injury	↓ Spinal cord trauma ↓ Neuronal apoptosis	Antioxidant Anti-inflammatory	[21–25]

Parkinson's disease	↓ Cognitive/locomotor defects ↓ Neuron loss	Antioxidant Anti-inflammatory	[8, 26, 27]

Diabetic peripheral neuropathy	Improved NCV ↓ Neuron loss ↓ Nontraumatic amputation	AMPK/PI3K Activation	[28, 29]

Diabetic/ischemic retinopathies	↓ Neuronal apoptosis Improved ERG ↓ Glial activation	Antioxidant Anti-inflammatory	[12, 30]

**Table 2:** Beneficial effects of PPAR*α* in microvascular disease and molecular mechanisms of action.

Model	Physiological effects	Molecular mechanism(s)	Ref(s)
Diabetic retinopathy	↓ EC dropout Improved pericyte survival ↓ Vascular permeability	Antioxidant Anti-inflammatory	[13, 15]

Peripheral neuropathy	↓ EC loss	↑ AMPK/PI3K signaling ↓ Endothelial dysfunction	[28]

Cerebral ischemia	↓ EC loss	Antioxidant Anti-inflammatory	[9, 19]

Type 2 diabetic patients	Improved endothelial function	eNOS activity Anti-inflammatory ↓ Dyslipidemia	[31–34]

Ischemic retinopathy	↓ Vascular permeability ↓ Retinal neovascularization ↓ EPC mobilization/homing	Anti-inflammatory ↓ Vascular growth factors ↓ EPC homing factors	[15, 35, 36]

Intestinal permeability	↓ Intestinal permeability	Tight junction protein localization	[37]

Nude mouse/wound healing	↓ Angiogenesis	↓ AKT signaling ↓ Vascular growth factors	[38, 39]
